# Reproductive Senescence Blunts Response of Estrogen Receptor-α Expression to Estrogen Treatment in Rat Post-Ischemic Cerebral Microvessels

**DOI:** 10.1371/journal.pone.0102194

**Published:** 2014-07-10

**Authors:** Emil Zeynalov, Niloofar Rezvani, Chikao Miyazaki, Xiaoguang Liu, Marguerite T. Littleton-Kearney

**Affiliations:** 1 Uniformed Services University of the Health Sciences, Bethesda, Maryland, United States of America; 2 Johns Hopkins University School of Medicine, Department of Anesthesiology and Critical Care Medicine, Baltimore, Maryland, United States of America; University of South Florida, United States of America

## Abstract

**Background:**

Several studies demonstrate that estrogen treatment improves cerebral blood flow in ischemic brain regions of young ovariectomized (OVX) rats. Estrogen receptor-α (ER-α) may mediate estrogen’s beneficial actions via its effects on the cerebral microvasculature. However, estrogen-derived benefit may be attenuated in aged, reproductively senescent (RS) rats. Our goal was to determine the effects of aging, estrogen deprivation and estrogen repletion with oral conjugated estrogens (CE) on postischemic cerebral microvascular protein expression of ER-α and ER-β.

**Methods:**

Fisher-344 (n = 37) female rats were randomly divided into the following groups: OVX, OVX CE-treated, RS untreated, and RS CE-treated. After 30 days pretreatment with CE (0.01 mg/kg) rats were subjected to15 min. transient global cerebral ischemia. Non-ischemic naïve, OVX and RS rats were used as controls. Expression of ER-α and ER-β in isolated cortical cerebral microvessels (20 to 100 µm in diameter) was assessed using Western blot and immunohistochemistry techniques.

**Results:**

Age and reproductive status blunted nonischemic ER-α expression in microvessels of OVX rats (0.31±0.05) and RS rats (0.33±0.06) compared to naïve rats (0.45±0.02). Postischemic microvascular expression of ER-α in OVX rats (0.01±0.0) was increased by CE treatment (0.04±0.01). Expression of ER-α in microvessels of RS rats (0.03±0.02) was unaffected by CE treatment (0.01±0.02). Western blot data are presented as a ratio of ER-α or ER-β proteins to β-actin and. Oral CE treatment had no effect on ER-β expression in postischemic microvessels of OVX and RS rats. Statistical analysis was performed by One-Way ANOVA and a Newman-Keuls or Student’s post-hoc test.

**Conclusion:**

Chronic treatment with CE increases ER-α but not ER-β expression in cerebral microvessels of OVX rats. Aging appears to reduce the normal ability of estrogen to increase ER-α expression in postischemic cerebral microvessels.

## Introduction

Brain ischemic stroke is a devastating neurologic disease associated with a high mortality rate and long-term disability in survivors [1], [2]. The epidemiology and severity of ischemic stroke varies in a gender- and age-specific fashion [1], [2]. Premenopausal and perimenopausal females have lower incidence of stroke than do age-matched males [2], [3]. However, the susceptibility of late post-menopausal women to stroke increases to a level beyond that of age-matched men [2], [3]. Data from both human and animal studies show that estrogen replacement therapy improves post-ischemic cerebral blood flow (CBF) in pre- and perimenopausal females, but the effect is lost with advancing age and prolonged estrogen deficit [2], [4]. Such observations have led to the concept that estrogen may act differently on the aging brain [3], [5]–[7] or that prolonged estrogen deprivation prior to initiation of estrogen replacement therapy may diminish the potential benefits of hormone therapy on brain rescue after an ischemic event. Several studies demonstrate that age can affect estrogen receptor (ER) distribution in the hippocampus, hypothalmus and cortex of old rodents [8]–[11], but the effects of aging on ER expression in the cerebral vasculature is unclear. It is possible that changes in cerebral microvascular ER expression or functionality may occur with aging or during periods of prolonged estrogen deprivation [12]. As a consequence, dysfunctional or diminished ER expression may depress the ability of the cerebral microvasculature to dynamically adjust blood flow to meet ischemic tissue needs. Ischemia-induced changes in cerebrovascular function as well as prolonged estrogen deprivation may exacerbate brain injury. In addition, age and prolonged menopause may be associated with impaired arterial function and diminished effectiveness of estrogen treatment [12]. We hypothesized that long-term estrogen depletion and/or aging may lead to alterations in ER expression in cerebral microvessels. Therefore, we assessed the effects of aging, estrogen deprivation and estrogen repletion with conjugated equine estrogens (CE) on cerebral microvascular expression of ER-α and ER-β after transient global cerebral ischemic injury.

## Methods

Experiments were carried out in accordance with the guidelines of the National Institutes of Health for the care and use of animals in research and were approved by Johns Hopkins University School of Medicine and Uniformed Services University of the Health Sciences Animal Care and Use Committees.

### Animal groups and experimental design

**Figure 1:**
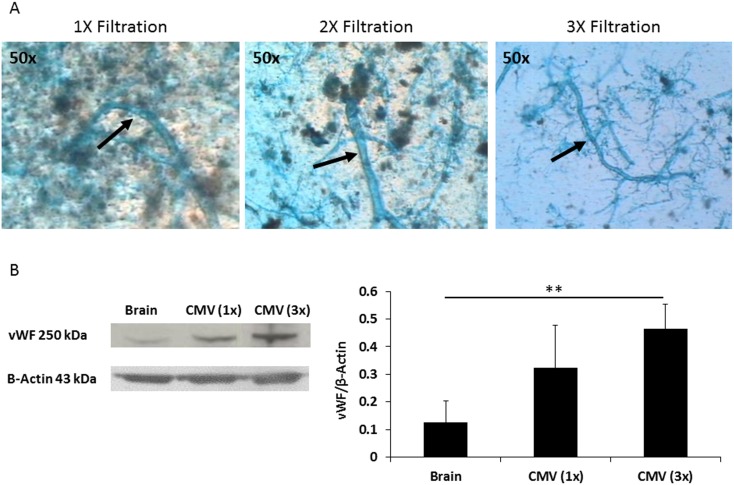
Filtration of cerebral microvessel samples improves purity. (A) Representative microscopy images (50×magnification) of suspended isolated cerebral microvessels stained with a 1% solution of Evans Blue in 0.9% NaCl. Arrows point to cerebral microvessels. Samples filtered three times through a 60-µm nylon mesh show substantially less cellular debris than those filtered one or two times. (B) Western blotting illustrates that the concentration of von Willebrand factor (vWF) protein increased significantly in samples filtered three times compared to samples filtered one time. Values of vWF expression are presented as a ratio to β-actin. All values are means ± SD; **p<0.01 vs. homogenized brain or cerebral microvessel samples filtered one time. Western blot data are based on three independent gels, with n = 3 per group.

We used 37 Fisher-344 female rats for this study: three-month-old (young) naïve, three-months-old ovariectomized (OVX), and 18–20-month-old (old) reproductively senescent (RS). For validation of the cerebral microvessel isolation technique 9 naïve female rats were randomly divided into 3 groups and used for Von Willebrand Factor (vWF protein specific to endothelial cells [13]) expression analysis: whole brain (n = 3), 1x filtration (n = 3), and 3x filtration (n = 3), Fig. 1. The other 28 animals were randomly divided into the following treatment groups: Naïve (n = 4), OVX pre-ischemic (n = 4), RS pre-ischemic (n = 4), OVX post-ischemic (n = 4), OVX+CE post-ischemic (n = 4), RS post-ischemic (n = 4), RS+CE post-ischemic (n = 4). In order to model surgical menopause young (three-months-old) rats were ovariectomized to decrease serum estrogen concentrations and to reduce associated hormonal fluctuations. The RS rats were left intact to model the normal reduction in estrogen and hormonal fluctuations associated with natural menopause. All rats were treated with 0.01 mg/kg estrogen (Premarin, Wyeth-Ayerst, Philadelphia, PA) daily for 30 days by the gavage method. Untreated OVX and RS rats were used as controls. After completion of estrogen treatment, all groups of rats were subjected to 15 minutes transient global ischemia using the 4 vessel occlusion method (see below). During all surgical procedures, rats were maintained under 1.0–1.5% isoflurane anesthesia mixed with 25% oxygen-enriched room air; all physiological variables were maintained within normal range.

### Ovariectomy

A 1-cm incision was made through the skin and muscle bilaterally approximately 0.5 cm below the edge of the rib cage. The ovaries were located and the ovarian arteries and veins were clamped and ligated. The ovaries were excised [14], and the abdominal muscle and skin were closed with 3.0 silk sutures.

### Transient global ischemia by the four-vessel occlusion technique

A midline incision was made at the base of the skull, the muscles were bluntly dissected, the *alar foramina* were visualized bilaterally and both vertebral arteries were cauterized. The common carotid arteries were dissected through a midline anterior neck incision. Ligatures were placed around both carotid arteries and were reversibly occluded for 15 minutes to induce 4 vessel ischemia as previously described [14]. After 15 minutes the ligatures were removed and the brain allowed to spontaneously reperfuse for 120 minutes. Body temperature, blood pressure, and arterial blood gases were maintained within the physiological range. Plasma estradiol concentration was evaluated at the end of the experiment by radioimmunoassay (Coat-a-Count; DPC, Los Angeles CA) as previously described [15].

### Isolation of cerebral microvessels

Cerebral microvessels were isolated as previously described [15] with modification. Each sample of microvessels was derived from the cortices of one rat brain. Immediately at the end of the experimental protocol, brains were perfused with normal saline and the cortex separated and manually homogenized in a glass homogenizer containing 3 mL of phosphate-buffered saline (PBS). Cellular debris was removed by centrifugation at 250 *g* (10 minutes, 4°C), and the homogenate was resuspended in 4 mL of 25% dextran (Sigma-Aldrich, Inc., St Louis, MO) in PBS. Samples were centrifuged at 2000 *g* for 20 minutes, and the supernatant was collected and passed through a 60-µm nylon mesh three times; samples were resuspended in 5 mL of PBS after each pass. The samples were stained with 1% Evans Blue and viewed through a light microscope to confirm successful isolation of the microvessels and to measure their diameter (Figure 1A). The PBS was removed, and the isolated microvessels were suspended in 0.5 mL of RIPA buffer (Sigma-Aldrich, Inc.) and homogenized by an ultrasonic homogenizer. Protein concentration was assessed by the Bicinchoninic (Smith) acid protein assay kit (Pierce, Rockford, IL).

### Immunohistochemistry

**Figure 2:**
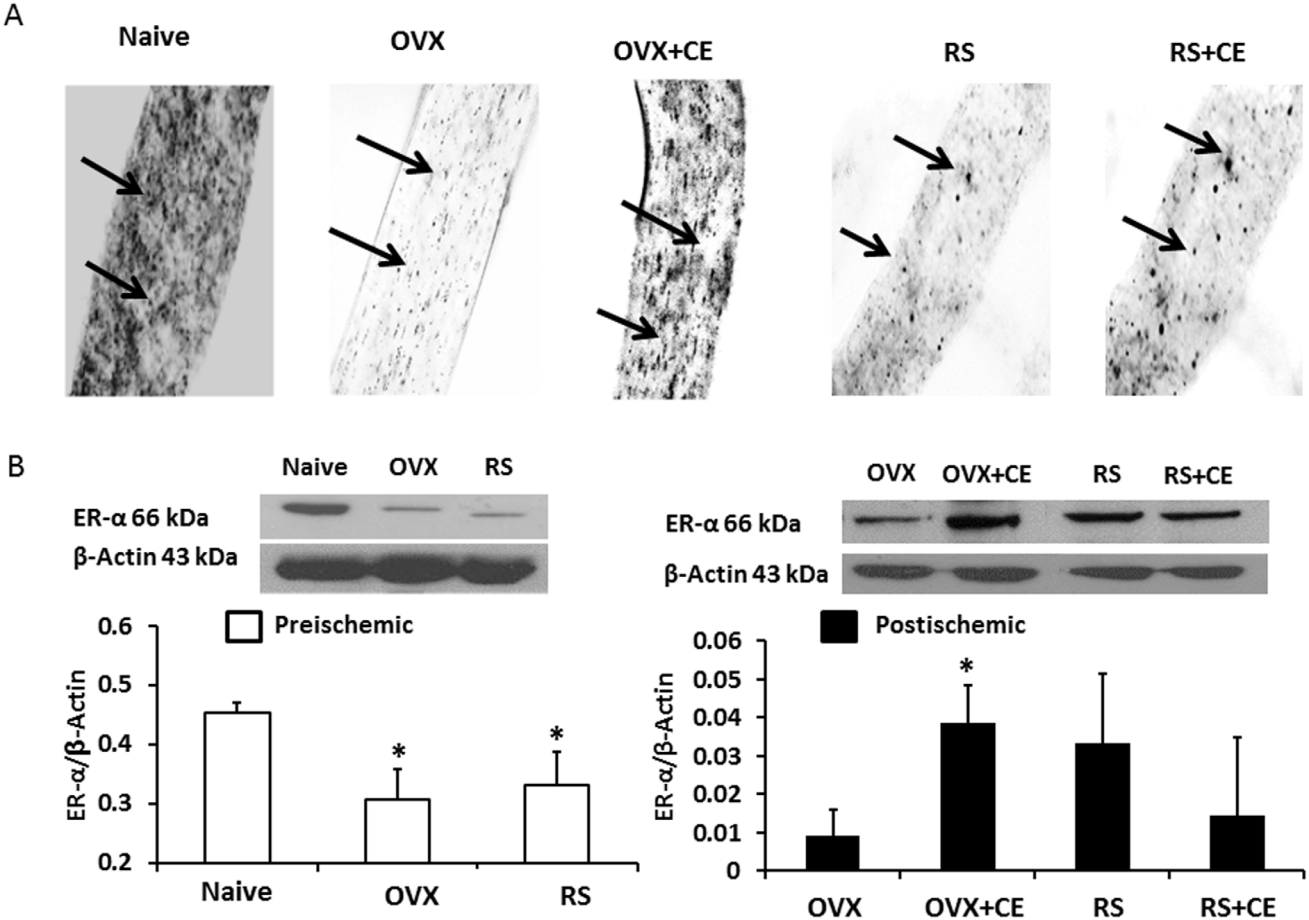
Representative slides obtained from immunohistochemistry staining (A) and Western blotting (B) demonstrate changes in ER-α protein expression in cerebral microvessels isolated from rats at baseline and at 2 hours of reperfusion. Young ovariectomized (OVX) and old reproductively senescent (RS) rats had lower levels of ER-α protein expression in the cerebral microvessels than did young, naive rats. Chronic oral conjugated equine estrogen (CE) treatment resulted in increased expression levels of ER-α protein in OVX, but not in RS rats. Arrows indicate ER-α protein (A). Values of ER-α are expressed as a ratio to β-actin and presented as mean ± SD; *p<0.05 vs. corresponding control. Data are from four independent gels, with n = 4 per group.

Isolated cerebral microvessels were fixed in 2% paraformaldehyde for 30 minutes and permeabilized with Triton X-100 (0.1%) for 5 minutes. Cerebral microvessels were suspended in 1% normal goat serum and then incubated with primary ER-α antibody (1∶5000, H-184, Santa Cruz Biotechnology, Santa Cruz, CA) overnight at 4°C. Cerebral microvessels were washed and incubated in the secondary antibody (1∶1000, BA-1000, Vector Laboratories, Burlingame, CA) for 2 hours at room temperature. Microvessels were suspended in ABC solution (Vectastain Kit, Vector Laboratories) for 2 hours at room temperature, and 3, 3′-diaminobenzidine (Sigma-Aldrich, Inc.) was used to identify the antibody complexes. Microvessels were mounted onto slides, dehydrated, and then covered with a glass cover slip for subsequent analysis. Light microscopy was used to visualize slides. Selected representative microvessels from each group were between 40 and 50 µm in diameter (Figure 2A).

### Western blot analysis

Western blot analysis was used to evaluate the expression of vWF, ER-α, and ER-β proteins in all treatment groups. Equal amounts of protein (10 µg per lane) combined with LDS Sample Buffer (Invitrogen, Carlsbad, CA) were loaded onto NuPAGE 4–12% Bis-Tris gels (Invitrogen). Samples from all groups were separated on the same gel to enable direct comparisons. Proteins were transferred onto nitrocellulose membrane with 0.2-mm pore size (Invitrogen) and incubated overnight at 4°C with primary antibodies for vWF (1∶100, A00082, Dako, Carpinteria, CA), ER-α (1∶1500, C-311, Santa Cruz Biotechnology), and ER-β (1∶200, 1531, Santa Cruz Biotechnology). Membranes were incubated with secondary antibodies for vWF and ERs (1∶5000, goat anti-mouse or anti-rabbit IgG-HPR, Santa Cruz Biotechnology) at room temperature for 90 minutes. Monoclonal primary mouse anti-actin antibody (1∶1000, Sigma) and secondary antibody (1∶10 000, goat anti-mouse IgG-HPR, Santa Cruz Biotechnology) were used as a loading control. Protein bands were detected by enhanced chemiluminescence reagents (ECL Western Blotting Reagents, Amersham, Piscataway, NJ) and exposure to Hyperfilm (Amersham). The band densities were analyzed by the MetaMorph software (version 7.1.0.0, 1992–2007). β-actin protein and background subtraction were used to correct band densities. All Western blots were analyzed based on three independent gels for vWF and ER-β, or four independent gels for ER-α expression evaluation. Western blot data are expressed as a ratio to β-actin.

### Statistics

Statistical analysis was carried out by one-way ANOVA with post-hoc Student-Newman-Keuls method or Student’s t-test; *p* values of <0.05 were considered significant. Western blot data for vWF, ER-α, and ER-β proteins are presented as a ratio to β-actin and expressed as means ± SD.

## Results

We examined serum estradiol levels in 3 month old young ovariectomized (OVX) and in 18–20 month old reproductively senescent (RS) female rats. Oral administration of CE (0.01 mg/kg Premarin, Wyeth-Ayerst, Philadelphia, PA) elevated serum estradiol concentration in OVX rats from 1.45±1.05 to 41.47±13.56 pg/mL and in RS rats from 10.28±2.95 to 59.45±27.29 pg/mL. All values are means ± SD (n = 4 per group).

### Isolation of cerebral microvessels

Microvessels were harvested from the cortex of OVX and RS rats. The diameter size of isolated cerebral microvessels in each sample ranged from 20 to 100 µm. The samples were filtered three times through a 60-µm nylon mesh to obtain a more purified collection of cerebral microvessels. This method resulted in cleaner samples containing less cellular debris than those filtered only once (Figure 1A). To verify the enrichment of microvessels in the filtered samples, we quantified von Willebrand factor (vWF) protein density using Western blot analysis. Data were obtained from three independent gels and expressed as a ratio to β-actin (43 kDa). Using this method we validated that the proportion of vWF (300 kDa) in the cerebral microvessel sample was serially increased in samples filtered three times (0.51±0.14) versus those filtered once (0.32±0.15) and compared to whole-brain homogenates (0.12±0.12; Figure 1B).

### Expression of ER-α protein in post-ischemic cerebral microvessels

We used Western blots to quantify ER-α and ER-β protein expression in isolated cortical microvessels. Cerebral microvessels isolated from OVX and RS rats before ischemia had a lower density of ER-α protein compared to those isolated from young naïve rats (Figure 2, A and B). As shown in figure 2, basal expression levels of ER-α protein (66 kDa) in microvessels from OVX rats (0.31±0.05) and RS rats (0.33±0.06) were similar and both were lower than in those from naive rats (0.45±0.02; *p*<0.05; Figure 2B, left panel). As expected, oral estrogen treatment increased postischemic microvascular expression of ER-α in the OVX+CE (0.04±0.01) compared to the OVX (0.01±0.02; p<0.05) rats (Figure 2B, right panel) at 2 hours of reperfusion. In contrast, chronic estrogen administration did not affect microvascular ER-α expression in the untreated rats RS 0.03±0.02 (0.03±0.02) compared to the treated RS+CE group (0.01±0.02) despite similar plasma estrogen levels between the young and the aged rats (Figure 2B, left panel). In addition, we sought to determine if we could visualize qualitative differences in ER-α microvascular expression from untreated OVX and RS rats compared to CE-treated animals. We observed a notable reduction in ER-α expression in vessels from both the un-treated and the CE-treated RS rats, but not in the young animals.

### Expression of ER-β protein in post-ischemic cerebral microvessels

**Figure 3:**
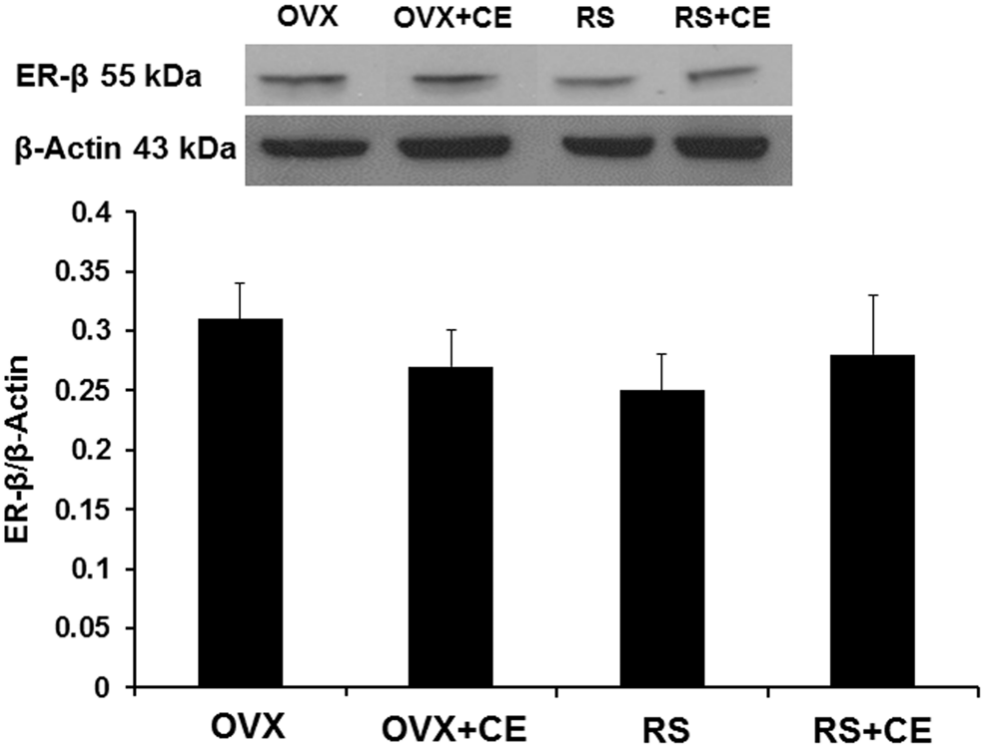
Estrogen treatment did not have any effect on ER-β expression in post-ischemic cerebral microvessels of young ovariectomized (OVX) or old reproductively senescent (RS) rats. Values of ER-β are expressed as a ratio to β-actin and presented as mean ± SD. Data are from three independent gels, with n = 3 per group.

Estrogen treatment had no effect on ER-β (56 kDa) expression in post-ischemic cerebral microvessels of OVX or RS rats. ER-β levels were 0.31±0.03 (OVX), 0.27±0.03 (OVX+CE), 0.25±0.03 (RS), and 0.28±0.05 (RS+CE) at 2 hours of reperfusion (Figure 3).

## Discussion

There are 3 principal findings of this study. First, age and reproductive status modulates baseline ER-α protein expression in cortical microvessels of young and aged reproductively senescent rats. Secondly, chronic treatment with oral conjugated equine estrogens alters the postischemic cerebral microvascular expression of ER-α protein in young OVX, but not in reproductive senescent rats. Whereas CE treatment up-regulated ER-α expression in the cerebral microvessels of young OVX rats, this effect was absent in the aged rats. Finally, administration of CE does not affect ER-β protein expression in cerebral microvessels of either young ovariectomized or aged reproductively senescent rats.

Very little is understood about the effects of aging and the concomitant reduction in plasma estrogen levels on estrogen receptors in the cerebral microvasculature. In the current study, we first show that estrogen depletion reduces ER-α expression in isolated cortical cerebral microvessels from non-ischemic young rats compared to naïve rats. Our data are consistent with others who also observed that ovariectomy reduces ER-α expression in cerebral microvessels of young rats [16]–[18]. We also demonstrate that estrogen depletion results in reduced ER-α expression in healthy 18–20 month old reproductively senescent rats similar to that observed in young ovariectomized rats. Even though we found slightly higher baseline plasma estrogen levels in the untreated RS compared the young OVX rats, we noted similar cortical microvascular ER-α expression. In contrast, Sandoval and Witt showed that the degree of cortical microvascular ER-α depletion was less pronounced in young ovariectomized compared to middle-aged (10–12 months old) rats [17] and this finding may be associated with higher plasma estrogen levels in middle-aged animals. Another study showed similar aortic ER-α expression in young naïve and aged (16 months old) spontaneously hypertensive rats (SHR) [19]. Estrogen levels in the aging rat fluctuate, but become lower and more stable between 16 and 22 months of age as the animals become acyclic [20]. The median life-span of F344 is approximately 24 months [21]. Based on these data, we believe that the RS rats in our sample are a good model of natural menopause. Further, in the F344 strain cessation of the estrous cycle occurs at approximately half of their life span, and this is similar to the timing of human menopause [22]. Therefore, a possible explanation for the differences in cerebral microvascular ER-α may be related to the fact that the rats in our studies were older with a presumably longer period of estrogen imbalance. Alternatively, target tissues may respond differently to estrogen loss and the cerebral arteries may be more sensitive than the aorta because of their differences in physiological specificity. For example, prolonged estrogen depletion reduced ER-α expression in the CA1 region of the hippocampus, but had no effect on uterine tissues [23]. These data paired with our observations suggest the possibility that varying estrogen levels affect estrogen receptor distribution and/or density differentially depending on age, and possibly localization (for example: cerebral vs. aortic arteries). Because women commonly receive oral estrogen replacement therapy and few pre-clinical studies have utilized the oral route, we chose to treat our rats with clinically relevant oral doses of CE (Premarin™). Drug dosages were calculated based on the normal daily dosages prescribed for postmenopausal women (CE = 0.625 mg). Previously, we showed that this dosage restores plasma estrogen to near physiologic levels [24] using the same dosing regimen. Oral CE administration resulted in a significant increase in plasma estrogen in both the young and the RS rats. We believe that differences we observed in cortical microvascular ER-α expression between the groups were unrelated to dissimilarities in plasma estrogen concentrations. However, the possibility exists that drug metabolism of young rats differs from that in old reproductively senescent rats [12] and this may contribute to the diminished postischemic ER-α expression in the RS compared to the young OVX rats that we observed. Further studies are needed to determine if estrogen metabolism is altered during aging.

Estrogen treatment produces a broad effect on multiple nuclear and non-nuclear pathways in blood vessels [25], [26]. In this study, we explored responses of ER-α and ER-β in post-ischemic cerebral microvessels after chronic oral estrogen treatment. However, recent studies report a third type of G-protein coupled estrogen receptor GPR-30 to be present in cerebral blood vessels [27]. GPR-30 is known to activate vasomotor responses of endothelial cells [27], but their relationship with exogenous estrogen or ER-α function has not been described yet. Future investigations are warranted in order to address whether or not chronic estrogen replacement impacts the function of GPR-30 in cerebral arteries.

Our data support that physiologic concentrations of estrogen have differential effects on ER-α expression in the postischemic cerebral microvasculature of young and aged rats. The importance of estrogen receptors in the postischemic brain remains unclear. However, existing data suggest the interactions of estrogen with its cognate receptors may be beneficial. During early post-ischemic conditions ER-α can maintain mitochondrial biogenesis [28], suppress free radical formation and lessen inflammation [28]. Estrogen has been shown to modulate endothelium-dependent dilation via processes that involve nitric oxide (NO), prostacyclin I_2_ and endothelium dependent hyperpolarizing factor [12], [29]–[31]. In the present study we chose to examine the effects of oral CE on the posti-ischemic cerebral vasculature because women commonly use oral estrogen replacement therapies. However, earlier clinical studies suggest that there are differential effects of oral over transdermal estrogen administration on endothelial function related to due to changes in vascular reactivity [32]. Interaction of the hormone with its cognate receptors may be important for normal vascular responses in the cerebral circulation. Findings of the present study suggest that prolonged estrogen abnormalities associated with aging may reduce the normal ability of estrogen to up-regulate ER-α protein expression in postischemic cerebral microvessels, but the mechanism of their loss with progression of age and reproductive senescence remains unexplained.

To our best knowledge, this is the first study to demonstrate that oral estrogen replacement has differential effects on ER-α expression in postischemic cerebral microvessels from aged compared to young rats. Although, involvement of ER-α in post ischemic vasoreactivity still remains unclear, we observed consistent increases in cerebral microvessels of estrogen-treated young OVX rats. Acyclic, reproductively senescent rats, however, seem unaffected by oral estrogen administration in terms of ER-α expression. Other investigations have documented changes in distribution of ERs in hippocampus and cortex in old rats [8]–[11]. In addition, prolonged periods of hypoestrogenicity are associated with hippocampal ER-α degradation subsequent to increased C-terminus of heat shock protein 70–interacting protein (CHIP)-induced proteosomal degradation [33]. Our current data extend these findings and show that aging alters the ability of pial arterioles to increase expression of estrogen receptor alpha. Our observations also suggest that estrogen deficit is linked to some degree of down-regulation of the cerebral microvascular ER-α expression (Fig. 2). The mechanism of this phenomenon remains unclear although the possibility exists that the receptor may also undergo age-induced structural changes leading to alteration in function that may be potentiated after ischemic injury. Future mechanistic studies of ERs in cerebral microvessels are indicated that may clarify functions of this important sex hormone receptor in cerebrovascular physiology, and its contribution to postischemic vascular recovery.

### Conclusions

Both young and reproductively senescent rats experience down-regulation of ER-α expression in cerebral microvessels with estrogen depletion. In young ovariectomized rats, estrogen replacement restores ER-α protein expression which is also sustained in postischemic conditions. Aged RS rats that have experienced presumably prolonged estrogen imbalance are unable to up-regulate ER-α protein in cerebral microvessels in response to estrogen treatment. In contrast, ER-β expression in cerebral microvessels seems to be unaffected by oral estrogen treatment in young and reproductively senescent rats. The specific mechanism underlying these age-related differences remains to be elucidated.
